# Changes in the Distribution of Atlantic Bluefin Tuna (*Thunnus thynnus*) in the Gulf of Maine 1979-2005

**DOI:** 10.1371/journal.pone.0075480

**Published:** 2013-09-19

**Authors:** Walter J. Golet, Benjamin Galuardi, Andrew B. Cooper, Molly E. Lutcavage

**Affiliations:** 1 School of Marine Sciences, University of Maine, Orono, Maine, United States of America; 2 Gulf of Maine Research Institute, Portland, Maine, United States of America; 3 Large Pelagics Research Center, UMass Amherst and Marine Fisheries Institute, Gloucester, Massachusetts, United States of America; 4 School of Resource and Environmental Management, Simon Fraser University, Burnaby, British Columbia, Canada; Aristotle University of Thessaloniki, Greece

## Abstract

The Gulf of Maine, NW Atlantic Ocean, is a productive, seasonal foraging ground for Atlantic bluefin tuna (*Thunnus thynnus*), but commercial landings of adult size classes were up to 40% below the allocated total allowable catch between 2004 to 2008 for the rod and reel, harpoon, and purse seine categories in the Gulf of Maine. Reduction in Atlantic bluefin tuna catches in the Gulf of Maine could represent a decline in spawning stock biomass, but given wide-ranging, complex migration patterns, and high energetic requirements, an alternative hypothesis is that their dispersal patterns shifted to regions with higher prey abundance or profitability, reducing availability to U.S. fishing fleets. This study fit generalized linear models to Atlantic bluefin tuna landings data collected from fishermen’s logbooks (1979-2005) as well as the distances between bluefin tuna schools and Atlantic herring ( *Clupea* *harengus* ), a primary prey species, to test alternative hypotheses for observed shifts in Atlantic bluefin tuna availability in the Gulf of Maine. For the bluefin model, landings varied by day of year, latitude and longitude. The effect of latitude differed by day of year and the effect of longitude differed by year. The distances between Atlantic bluefin tuna schools and Atlantic herring schools were significantly smaller (p<0.05) than would be expected from a randomly distributed population. A time series of average bluefin tuna school positions was positively correlated with the average number of herring captured per tow on Georges Bank in spring and autumn surveys respectively (p<0.01, r^2^=0.24, p<0.01, r^2^=0.42). Fishermen’s logbooks contributed novel spatial and temporal information towards testing these hypotheses for the bluefin tuna fishery.

## Introduction

In summer and autumn, schools of Atlantic bluefin tuna (*Thunnus thynnus*) (ABFT) enter the Gulf of Maine and adjacent regions to forage on lipid-rich herring and other prey before dispersing offshore and southward during winter and spring [1,2]. Atlantic bluefin tuna and their principal prey, Atlantic herring ( *Clupea* *harengus* ), support valuable commercial fisheries that have operated for decades in New England and the Canadian Maritimes [3,4]. From 1980 to 2003, the commercial ABFT fleet landed its total allowable catch (TAC). From 2004-2008, commercial landings (fish >185cm curved fork length, CFL) of ABFT in the Gulf of Maine declined to approximately 40% of levels recorded in the previous two decades [5,6]. The decline in U.S. commercial ABFT catches has, in part, been used to support a proposal to list this species under Appendix I of the Convention on International Trade in Endangered Species (CITES). Organizations, (e.g., U.S. Fish and Wildlife Service and the Committee on the Status of Endangered Wildlife in Canada) have conducted an evaluation for this species and list them as a “species of concern” and “endangered” respectively [7,8]. Given that severe population declines are a prerequisite for endangered species status of CITES approval, these reviews endorsed a range contraction hypothesis [9]. However, the most recent ABFT stock assessment indicates spawning stock biomass has been stable between the mid-1980’s to the mid-2000’s and increased slightly over the past few years [5] indicating alternative hypotheses may explain changes in U.S. commercial landings of ABFT [6].

Single species assessment approaches for highly migratory species focus primarily on dynamics of the target fisheries, and utilize catch rates (catch per unit effort) as a proxy of true abundance. At smaller scales, such as the northwest Atlantic shelf, utilizing CPUE without consideration of catch location or ecosystem state can be problematic when interpreting declines in landings for highly migratory species. For example, U.S. jurisdictional waters in the Gulf of Maine extend 200 nautical miles from shore. Given the migratory capacity of ABFT, relatively small shifts in their spatial distribution can place them across international boundaries where they cannot be pursued by U.S. fishermen. This change in spatial distribution does not necessarily reflect a change in abundance, but could highlight their responses to changing environmental conditions as observed in multiple groundfish species in this region [10].

For top predators, such as tunas, biophysical factors and behavior may also contribute to changes in their distribution. For example, food habit studies have highlighted the importance of ABFT predator-prey relationships in the Gulf of Maine [1,11-14] while direct observation of schools via aerial surveys portrayed real-time links between school location and biophysical features [15,16]. Bluefin tuna schools often exhibit clumped distributions, high inter-annual variability [17,18], and proximity to sea surface temperature (SST) fronts [15,16]. Dispersal patterns monitored over short (48 h) periods using hydro-acoustic tracking [19] and longer durations (up to one year) from popup satellite archival tags, exhibited high variability both in regional and Atlantic-wide movements [2,20-22]. In the Gulf of Maine, ABFT schools were associated with herring schools [23] and SST fronts [15], while modeling of movements based on ~48h telemetry tracks predicted prey patch size and distribution consistent with deck based observations [24]. Despite these informative insights, none of these data sets provide spatial information on catches spanning multiple years, nor do so at any point during the period of decline (2004-2008) for the US commercial ABFT fishery. As such they are of limited utility in current ABFT stock assessments or to explain changes in landings.

Characterized by large size [11], long life span [25-27], wide thermal and depth range (3-30°C, 0 > 1000m) [2,19,20,22,28-30], endothermic capacity [31-34], and diverse food habits [1,11-14], the ABFT’s eco-physiological traits likely contribute to complexity in its population dynamics [35-37], but ecosystem impacts are still mainly surmised. For example, catches monitored for centuries in Mediterranean fish traps exhibit frequency patterns of 20 and 100 years, with abundance varying by nearly an order of magnitude [38]. To date, fishery-dependent indices of abundance are the main source of information used in current ABFT stock assessment models [5,39], but alternative information describing dispersal patterns [19,20,22,29,30] and availability to fleets should be included, particularly since CPUE data alone do not adequately represent real abundance [40-42].

Two hypotheses, reduced availability of ABFT in U.S. waters due to a shift in spatial distribution, and range contraction as a result of reduced western Atlantic spawning stock biomass, have been proposed to explain declines in US commercial catches [6]. Support for either scenario or the exploration of alternatives has yet to be fully explored.

Spatially explicit information on the distribution/landings of ABFT in the Gulf of Maine is limited because landed fish are assigned to large statistical areas (1000’s kilometers) inadequate to discern changes in distribution on smaller scales (10’s to 100’s kilometers). However, commercial ABFT fishermen usually maintain detailed log books of their observations and catches. This study uses datasets from commercial fishermen’s logbooks to examine the distribution and availability of large, commercial sized (>185 cm CFL) ABFT in the Gulf of Maine across three decades, and explores potential biological drivers of inter-annual variability via the relationship of ABFT with Atlantic herring, their principle prey in this ecosystem.

## Materials and Methods

### Atlantic Bluefin Tuna Dataset

We collected fishery dependent data (1979-2005) from captains’ logbooks across commercial fishing categories (purse seine, harpoon, and rod and reel) to create a time series of high resolution information for ABFT in the Gulf of Maine. In logbooks, vessel captains recorded date, time of day, position (latitude and longitude or Loran C), and (usually) number of fish observed in the school and/or captured by the vessel. Data from captains’ logs were transcribed into an electronic spreadsheet and where applicable, Loran C (time distance) locations were converted to latitude and longitude using the Loran GPS Pro software (Arden Software Co. Indialantic, FL), (accurate to 0.2 microseconds). The final dataset contained 671 individual schools (> two fish) and a total of 46,350 individual ABFT observed or captured from 1979-2005. All fish were presumed or measured to be > 185 cm CFL, the minimum size for commercial landings. Vessel logbooks were personal records which were not required by federal or state agencies. These were personal logs were used by captains to monitor their previous and current catches. In 1989, the state of Massachusetts did require purse seine vessels to submit catch reports which indicated the number of fish captured and the location of those catches. We used these two sources of information to validate the location of catch locations from personal logbooks by checking the positions against the state required catch reports for purse seine catches. Inconsistencies in catch locations between personal logbooks and the state mandated catch reports if present were excluded. In addition, aerial surveys conducted between 1993 and 1997 by ABFT spotter pilots from the commercial fishery verified the location of surface schools via GPS receivers [17]. Some of the observed schools were subsequently captured by the commercial fleet which allowed cross validation of commercial logbook data. No changes in the reporting requirements for the state of Massachusetts were observed from 1989 to the end of the time series in 2005.

### Atlantic Herring Dataset

Commercial fishery information recorded since 1960 by the Maine Department of Marine Resources consists of landing dates, catch weight in metric tons, gear type, and location. From 1960 to 1995 catch reports were aggregated by 10 minute squares (latitude and longitude) and consequently, the spatial scale for this period was not useful for our analysis and was excluded. In 1996, commercial vessels submitted trip reports (VTR’s) that recorded each catch position, providing a 12 year (1996-2007) time series of herring catches in the Gulf of Maine of similar spatial resolution to our ABFT dataset. For herring data, we included only catches from purse seine and trawl gear types, in fishing zones 1A, 1B and 3 (Gulf of Maine and Georges Bank). This accounted for >95% of landings in these areas, where spatial overlap between ABFT and Atlantic herring fisheries exist. The final dataset contained 8,487 positions of Atlantic herring catches. From the National Marine Fisheries Service biannual bottom trawl surveys (for Atlantic herring and other commercially important species in the Gulf of Maine), we used the mean number of herring/tow as a proxy for the abundance of Atlantic herring in offshore strata including Georges Bank. We conducted correlation analysis between this time series and the weighted average position of ABFT schools.

### Model Selection for Atlantic Bluefin Tuna and Atlantic Herring

A generalized linear model (glm) with Poisson errors was fit to the ABFT catch data using the statistical package R [43]. The full model for ABFT consisted of four variables and six interactions as follows: glm(numbersoffish~(year) +(dayofyear) +(latitude) +(longitude) +(latitude*longitude) +(latitude*dayofyear) +(longitude*dayofyear) +(latitude*year) +(longitude*year)). Numbers of fish represents the number of fish observed or captured and day of year represents time from June 1^st^ to October 31^st^. The original fit with Possoin errors indicated over dispersion (residual deviance/degrees of freedom >1.5), so the model was refit using a quasi-Poisson distribution. Model simplification was conducted using a backwards step-wise variable selection reduction. We removed non-significant variables (p>0.05) starting with the interaction terms and working down to main effects, iteratively re-estimating model fits.

### Kernel Density Estimation

An interpolated surface density of Atlantic herring was created using non-parametric density estimators. Catch locations describe finite points which do not represent the extent of herring school distribution. Interpolated densities spread out this distribution and provided a more realistic distribution of the schools. We used two-dimensional kernel density estimation with an axis-aligned bi-variate normal kernel, evaluated on a customized 500 by 500 square grid (representative of the Gulf of Maine). The boundaries of the grids were defined based on the spatial distribution of catches within U.S. waters and were bounded by 39° N to 45° N and 71° W to 65° W. Kernels were created using the kde2d function within the MASS [44] library in the statistical package R [43]. Kernels were weighted by the metric tons of catch from an individual trip. We used the most common form of density estimation: where *x*_*1*_……..*x*_*n*_ are samples, *K* is a fixed kernel with a bandwidth *b*. Optimal bandwidth was determined using the bandwidth. nrd function which uses a well-supported rule for finding the optimal bandwidth utilizing the 25^th^ and 75^th^ distribution quantiles [44]. Kernels for the Atlantic herring dataset were computed at two week intervals.

### Atlantic Bluefin Tuna and Atlantic Herring Spatial Relationships

Resource selection models often use 95^th^ percentile contours from kernel density estimators to examine utilization distributions [45]. To assess the spatial relationship between Atlantic herring and ABFT schools, we compared distances from observed ABFT schools to the nearest 95^th^ percentile contour of the herring catch distributions (above) and distances from randomly distributed points to the nearest 95^th^ percentile contour. Geodesic distances were used to account for spatial curvature [46]. A 2:1 (random to actual) ratio was used for each two-week period. A bathymetry filter was used to prevent placing randomly generated points in areas ABFT would not be found (e.g., estuaries, rivers). A linear model was used to determine whether observed ABFT schools were significantly closer (alpha = 0.05) to herring distributions than one would expect if ABFT schools were randomly distributed (as represented by the random locations).

## Results

### The most parsimonious ABFT model contained three variables and four interactions

**Figure 1:**
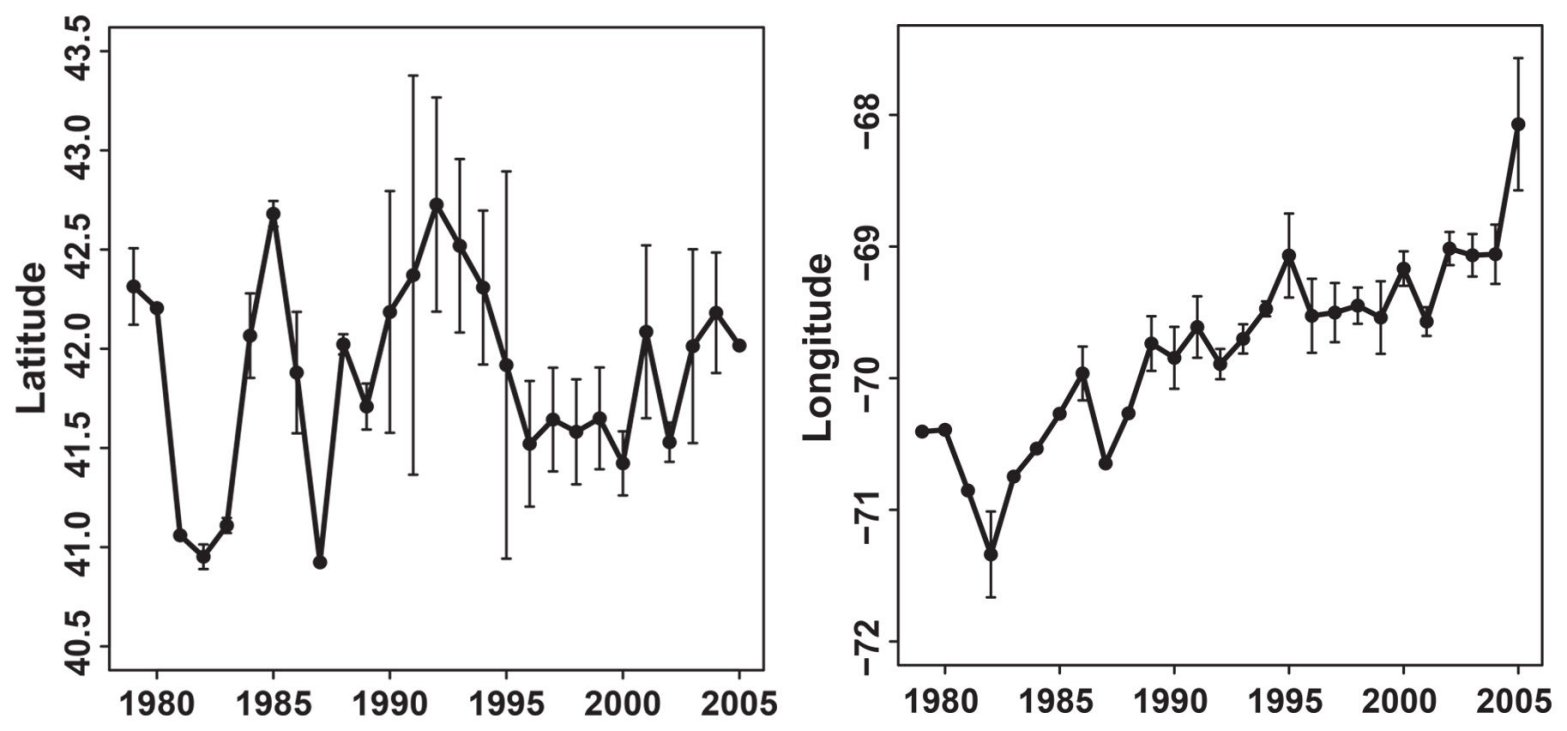
Surface school positions of Atlantic bluefin tuna in the Gulf of Maine. Each location represents the mean position of bluefin schools observed by spotter pilots, from vessels or represents captured fish from the commercial fishery. Within each year, mean positions were weighted by the number of fish in each school. Error bars represent sample variance. Schools of Atlantic bluefin tuna have shifted their longitudinal distribution approximately 280km to the east (70.95 to 68.47° W).

**Figure 2:**
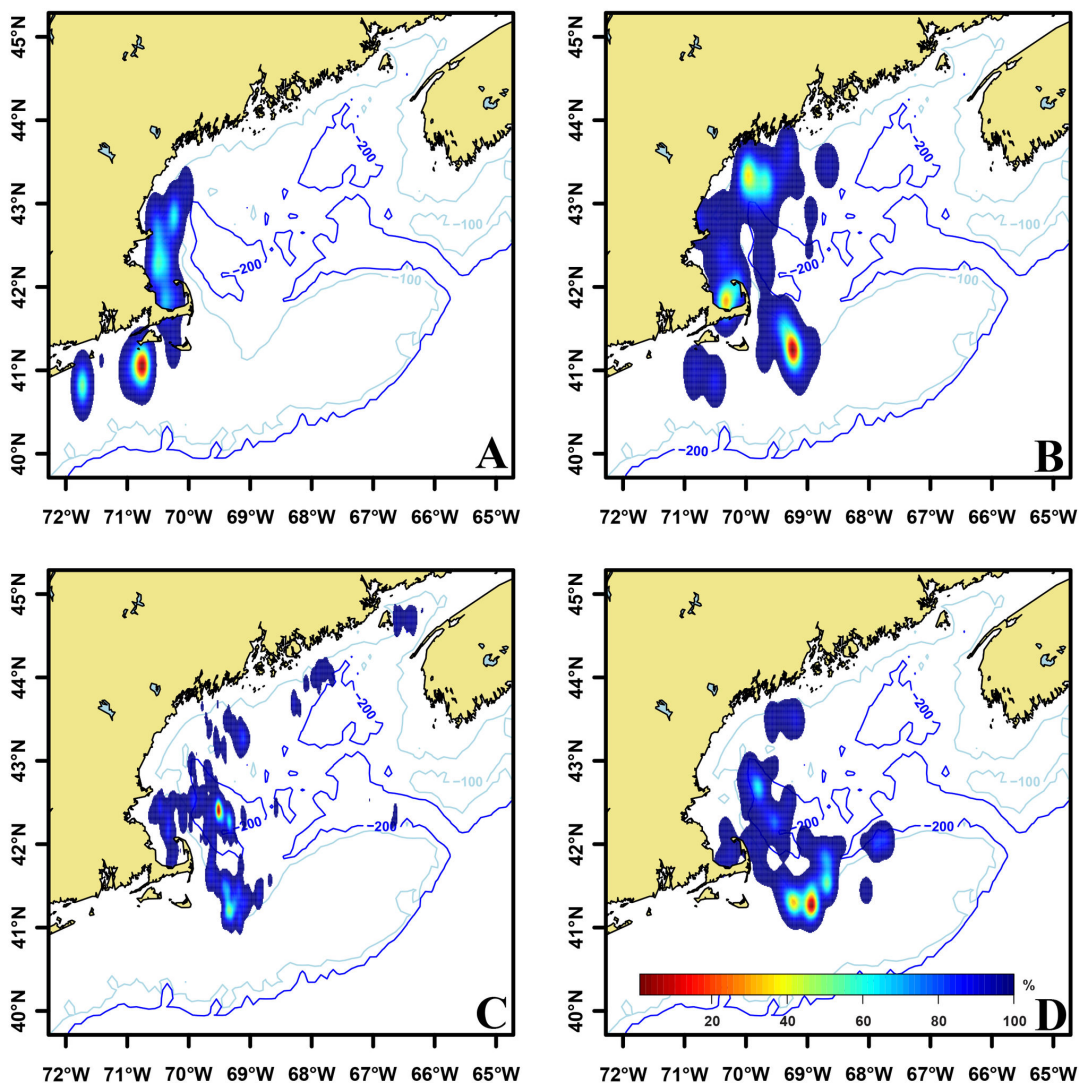
Distribution of Atlantic bluefin tuna in the Gulf of Maine. Kernel density estimations were constructed based on the number of fish observed in each school over four selected time periods. Density estimations were normalized yielding utilization distributions that displayed probability of occurrence during four time periods, A) 1979-1985, B) 1986-1992, C) 1993-1999, and D) 2000-2005.

**Figure 3:**
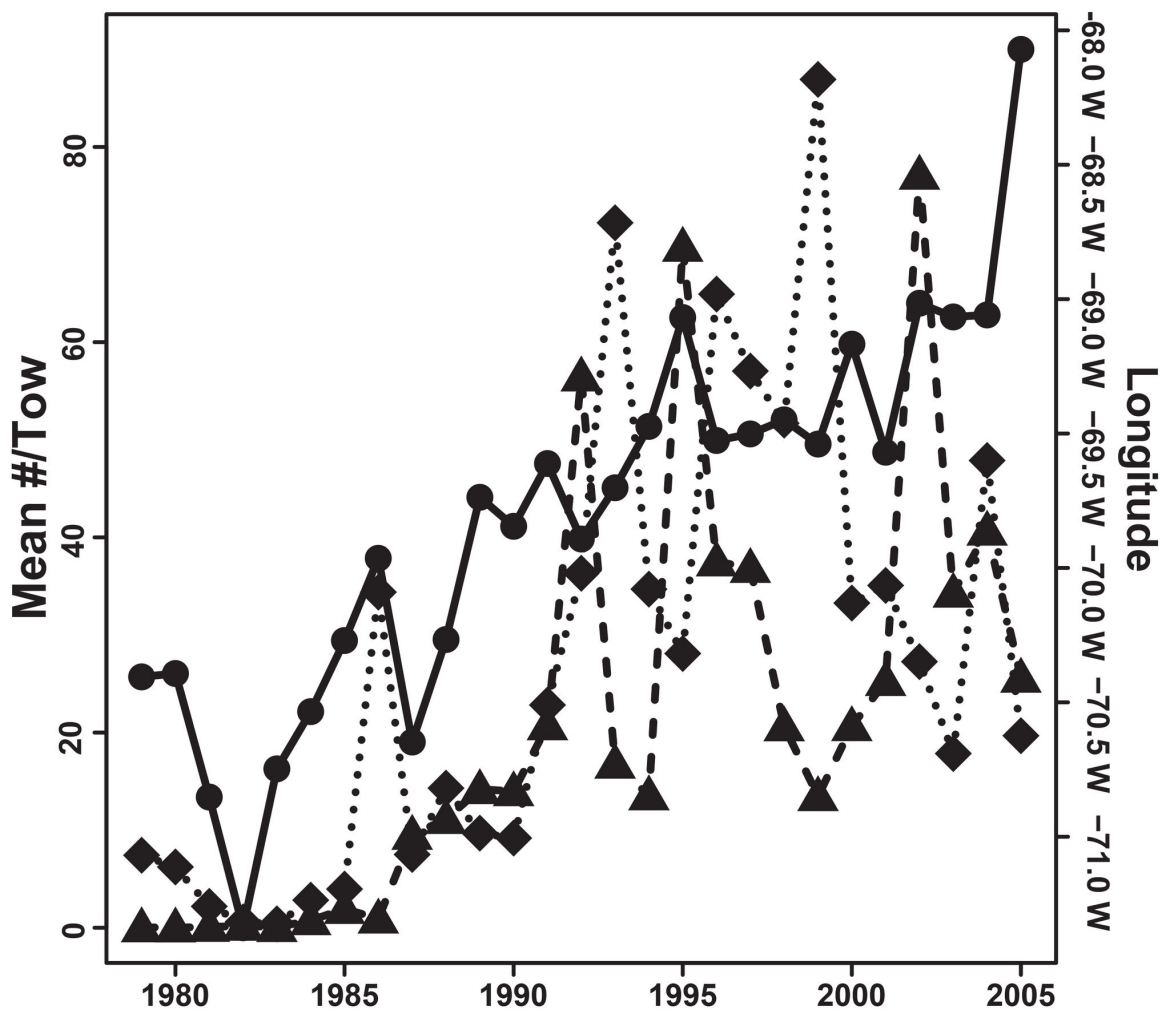
Average number of Atlantic herring/tow and position of Atlantic bluefin tuna schools. Each bluefin tuna point represents a weighted mean of surface schools (solid black circles) and is correlated with an increase in herring/tow on Georges Bank during the spring (solid black triangles) and fall trawl survey (solid black diamonds). Atlantic herring data represented as the mean herring per tow from the (National Marine Fisheries Service) autumn bottom trawl survey for survey strata on George’s Bank.

glm(numbersoffish)~(dayofyear)+(latitude)+(longitude)+(dayofyear) +(latitude*dayof year) +(latitude*year) +(longitude*year)). Three variables, (day of year, latitude and longitude) and two interactions (latitude* day of year and longitude * year) were significant (p<0.05) in the ABFT model. The effect of latitude differed depending on the day of year and the effect of longitude differed across years. Average latitude of ABFT schools in the Gulf of Maine ranged from 40.95 to 42.72°N with no north or south trend (Figure 1). Average longitude of ABFT schools shifted east > 280 kilometers (-70.95 to -68.47° W) and exhibited high inter-annual variability (Figure 1). Intra-annual distribution of schools was variable. Some years contained one or two high density aggregations while others had multiple high density aggregations distributed across a broad spatial range (Figure 2). There was a significant correlation (p<0.05) between the average position of ABFT schools and the average Atlantic herring/tow in both spring (p<0.01, r^2^=0.24) and autumn (p<0.01, r^2^=0.42) surveys from offshore strata, including Georges Bank (Figure 3).

**Figure 4:**
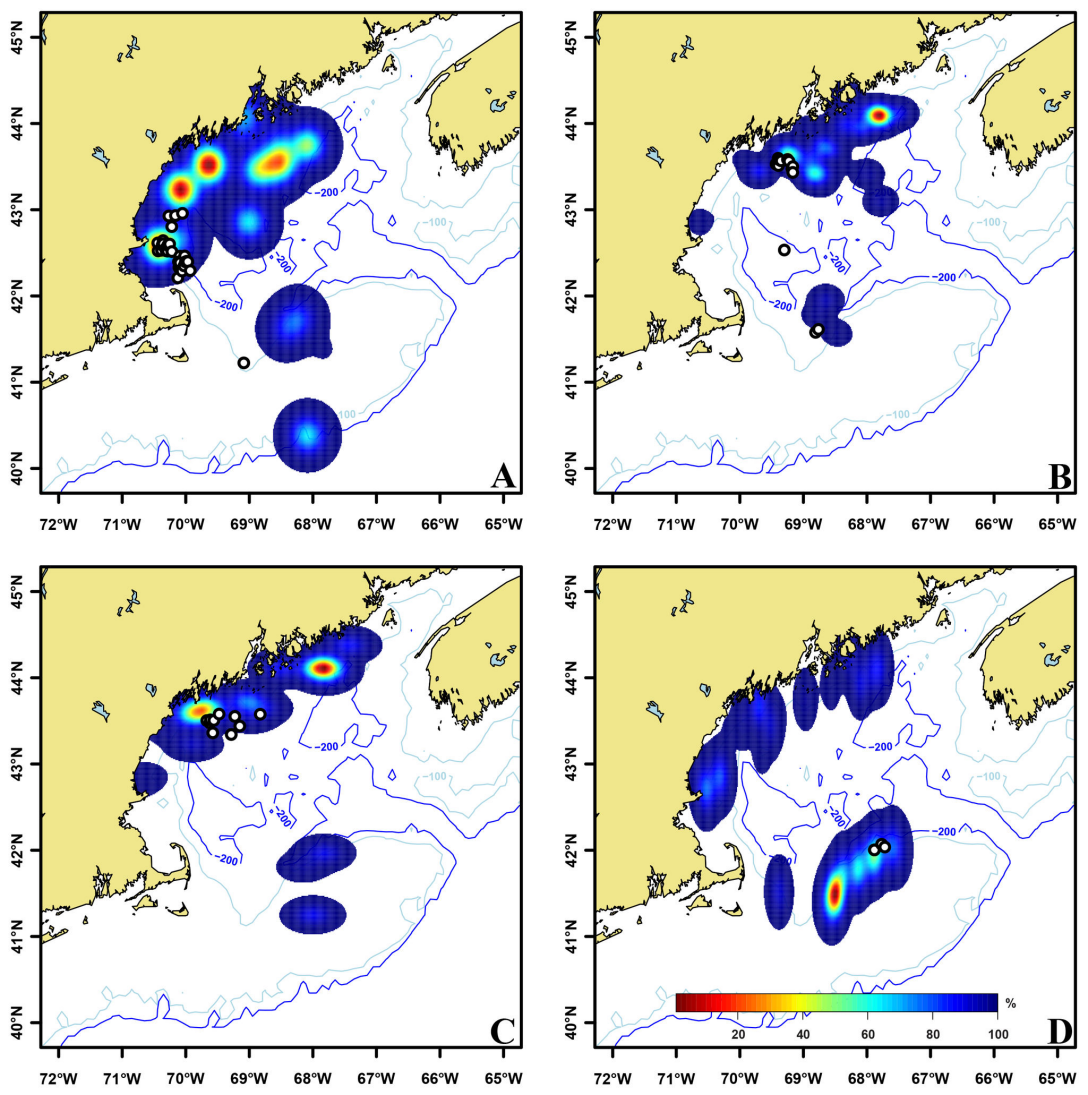
Spatial relationship between Atlantic herring and Atlantic bluefin tuna in the Gulf of Maine. Atlantic herring kernel density estimations were constructed based on the metric tons of herring captured in each tow or set (trawler or purse seiner, respectively). Density estimations were normalized yielding utilization distributions that displayed probability of occurrence during four two week time periods, A) June 1-15 1997, B) August 16-30 2003, C) July 16-31 2004, and August 1-15 2005. Atlantic bluefin tuna (white circles with solid black borders) were located closer to high probability regions of Atlantic herring than a randomly distributed population. While the majority of bluefin tuna schools were located within areas of high herring probability, some schools of bluefin tuna did not display any association with herring.

Significant differences were observed between those distances calculated from the 95^th^ percentile utilization distributions of Atlantic herring catch locations, position of ABFT surface schools and the randomly generated ABFT surface schools (p<0.05). Results identified schools of ABFT were located closer to high densities of Atlantic herring than a population distributed at random. From a total of one hundred two-week time periods between June 1 and October 31 1996-2005, ABFT were observed within the 95^th^ percentile utilization distribution of Atlantic herring 17 times (Figure 4). The remaining ABFT schools were located inside or along the margin of the 50^th^ percentile utilization, or beyond areas of high herring density (Figure 3).

## Discussion

For our analysis we used data derived from fishermen’s logbooks to create the first long-term, multi-decadal, spatially explicit time series of commercial ABFT catches and sightings in the Gulf of Maine. Our results portray the distribution of commercial size ABFT schools (>185 cm CFL) and provide evidence that a gradual longitudinal shift to the east has reduced availability to the U.S. fishery and contributed to the observed decrease in U.S. commercial landing in the Gulf of Maine. Fishermen’s logbooks have been used to interpret and improve the quality of information used in stock assessments [47] and provide additional context for evaluating regional shifts in CPUE- based indices of abundance. This work provides an example of how fishermen’s records can provide additional information beyond that contained within traditional stock assessment usage. When utilizing fishermen’s knowledge, it is important to identify the nature and limitations of logbook-derived observations [48,49], and to recognize that fishing effort, like CPUE, is affected by multiple factors. Purse seine and harpoon vessels use spotter pilots (and fish sounders) to locate ABFT schools, but the amount of search conducted by the vessel and spotter plane is highly variable [16,18]. Purse seiners target surface schools containing tens to hundreds of individuals, and cease fishing when the hold is full, target catch has been landed, or fishing conditions deteriorate. Harpoon boats target fish until a bag limit or capacity is reached, or when fishing conditions are no longer suitable. Based on fleet dynamic models [50], we presumed that fishermen in our study would fish areas closest to shore where catch is profitable, and adjust fishing effort relative to perceived fish distribution and quality.

Despite its multi-decadal length and broader set of observations, our dataset shares some limitations common to catch-dependent information. Our data-set is biased by the search area of vessels and spotter planes and cannot account for areas not surveyed by the fleet, nor can it be used to interpret the presence or absence of fish in other regions. However, it is of appropriate temporal-spatial scale for examining the distribution of ABFT over the extent of the commercial fleet’s range.

Over 28 years, the distribution of ABFT in the Gulf of Maine shifted steadily eastward by >280 km (i.e. about 2.5 degree longitude). This shift in distribution took place despite a period of high seasonal abundance (1993-1996), when aerial surveys documented hundreds of surface schools and tens of thousands of individuals of commercial size were present at the surface alone in the western and central Gulf of Maine [17,18]. By 2005-2008, U.S. commercial ABFT catches dropped sharply [51,52]. Shifts in ABFT distribution and relative abundance have been documented throughout their range, over decades to centuries [38] and across broad areas [53,54]. The decline in Nordic ABFT fisheries was attributed to overfishing, recruitment failure (i.e., increasing mean size of catches), and overfishing of prey [53,55-58]; reasons advanced for their disappearance off Brazil in the 1960’s include over-exploitation, changing oceanographic regimes, and adoption of different migratory pathways [57,59].

Changes in U.S commercial landings may also be explained by different levels of mixing between the two stocks. Recently, biomarker studies indicated that large contributions of small, eastern origin fish are present on western Atlantic foraging grounds. Though mixing between eastern and western stocks is well established [20,22,29,30] otolith microchemistry suggests that commercial size ABFT sampled in the Gulf of Maine are almost exclusively from the western stock [60]. This indicates that substantial contributions of eastern fish are not supporting U.S. commercial ABFT fisheries in the Gulf of Maine, at least, not over the recent period of observation and biological sampling. From 1994 to 2005 the average length of commercially landed ABFT in the Gulf of Maine increased by 35 cm, a scenario similar to the catch history of the Nordic fishery, and could be interpreted as recruitment failure. However, an influx of juvenile ABFT from an apparently strong 2003 year class [51,52], indicated that fish are still recruiting to the region. Nonetheless, lower catch rates from 2005-2008 could have resulted from an anomalously long gap between successful year classes of ABFT. As the strong year classes of the 1990’s continued to grow and exit the fishery, subsequent year classes may not have been sufficient to sustain commercial catch levels. However, historically high catches in offshore U.S. and Canadian shelf areas do not support this hypothesis [5]. Declines in U.S. commercial landings may also be related to an observed decrease in condition and lipid reserves [61] which could reduce reproductive output [62] and affect recruitment, but no changes were detected in ABFT larval surveys conducted in the Gulf of Mexico [5]. Since early life history of ABFT in the western Atlantic is poorly described, and young of the year (Mather’s “cryptic biomass” [3] are rarely encountered, this hypothesis is difficult to resolve.

**Figure 5:**
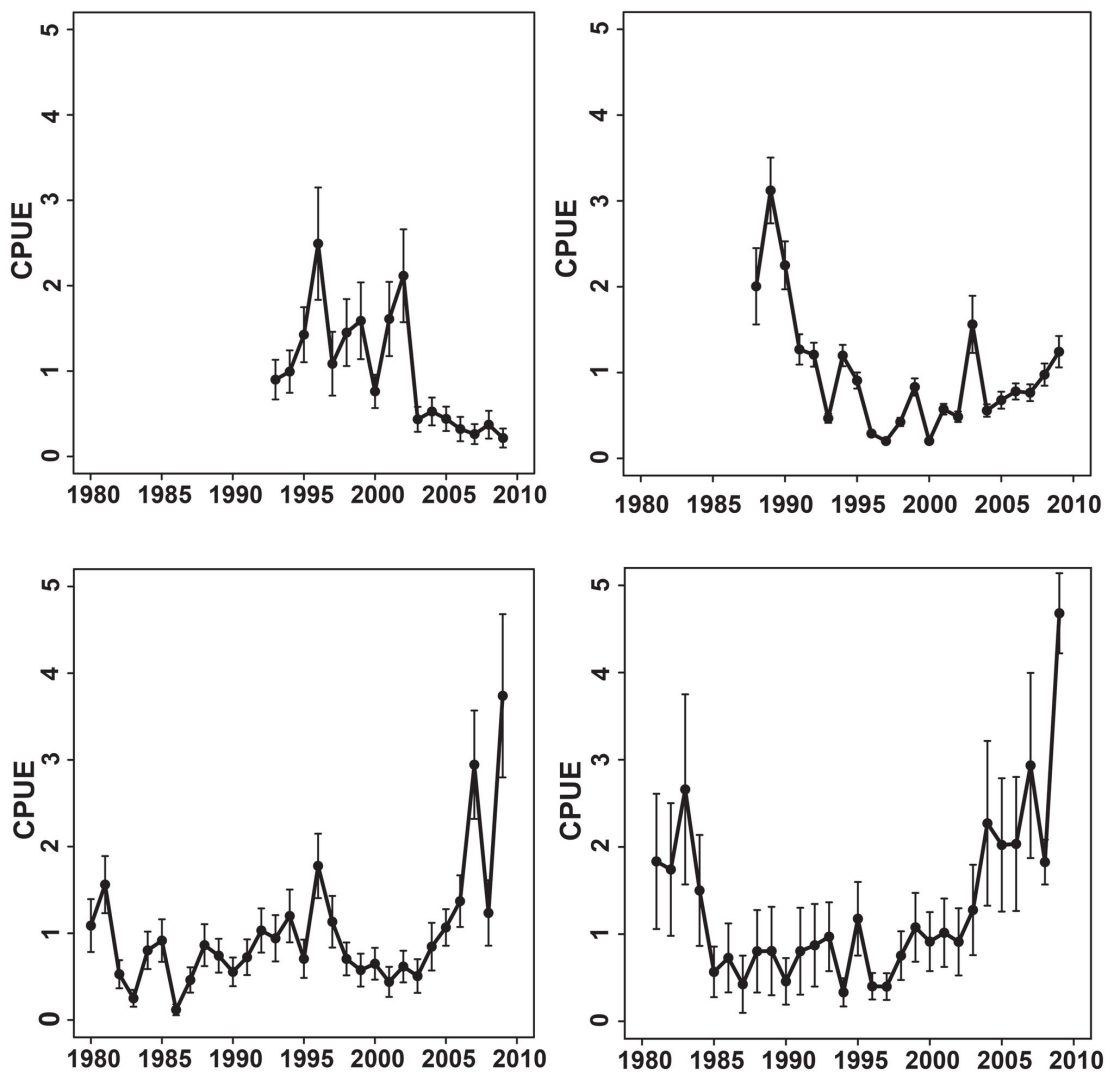
Catch per unit effort (CPUE) for Atlantic bluefin tuna calculated across four regions of the northwest Atlantic shelf. (A) The Gulf of Maine CPUE has been in decline since 1996. Adjacent shelf regions such as (B) southwest Nova Scotia, (C) the offshore northwest Atlantic area two, and (D) the Gulf of St. Lawrence all show increasing catch per unit effort during periods of decline in Gulf of Maine.

Concomitant with the declines in Gulf of Maine CPUE, catches of ABFT in adjacent Canadian and Japanese longline fisheries (e.g., Scotian Shelf and Gulf of St. Lawrence) have increased [5] (Figure 5). Such increases could be explained by the occupancy-abundance relationship, where fewer individuals occupy only the most profitable foraging habitat (hyper-aggregation), and reductions in CPUE within the Gulf of Maine are mainly due to reduced spawning stock biomass of ABFT. While one study suggested that range contraction has occurred from overfishing [9], this is not supported by Atlantic wide catches or fishery-independent results from electronic tagging [20-22,29,30]. Alternatively, the length based migration hypothesis [63], stable fishing mortality [5], increasing spawning stock biomass [5], increasing CPUE in adjacent fisheries [5,64], and lack of decline in the US larval survey [5] do not generally support this and, alternatively, points to ecosystem changes as a possible cause of the decline in US commercial landings.

Declines in ABFT landings over broad geographic regions have been linked to environment [65,66] and, while making such connections on smaller scales has proven more difficult [16,67], prey is presumed to be the principle driver [23]. In the Gulf of Maine, recent shifts in size classes from mostly “giants” to smaller/younger ABFT, and differences in their preferred prey (herring vs sand lance, respectively) [13,14], may indicate that there was insufficient prey biomass to meet bio-energetic needs of the adults. However, current spawning stock biomass of Atlantic herring in the Gulf of Maine and Georges Bank is high [68] which suggests that prey biomass itself is not regulating this shift, or that there are distinct differences in the abundance of inshore and offshore herring assemblages [68]. Life history models show that ABFT need to maintain sufficient energy stores in order to spawn annually [62] and thus, over a long lifespan, should seek forage grounds and shorter migration paths with the highest energetic returns. Declines in somatic condition of ABFT during the 1990’s [61] may indicate that diet profitability in the Gulf of Maine was not capable of maintaining sufficient lipid levels for successful ABFT migration and reproduction. The rapid rebuilding of the Gulf of Maine Atlantic herring stock and the disproportionate herring abundance between Georges Bank and inshore Gulf of Maine (80% of which is located on Georges Bank) [68,69], would make the offshore areas more profitable for ABFT. While the significant relationship between the shifts in ABFT distribution and the average number of herring per tow in autumn bottom trawl surveys suggest ABFT moved in response to a higher level of herring abundance on Georges Bank, herring abundance alone cannot explain this shift. While lipid reserves declined in adult ABFT [61], coincident biophysical changes occurred in the Gulf of Maine during the 1990’s [70-72], altering patterns of groundfish recruitment as well as phytoplankton and zooplankton communities [70-75]. The reasons for these lipid declines could only be inferred because ABFT spend only about a third of their annual migration cycle in northern feeding areas [1,76]. While some individuals continue to forage off the Carolinas and mid-Atlantic shelf [2,77] after leaving the Gulf of Maine, possibly to “top off” energy stores, others migrate offshore [21,22,29,30].

Since ABFT forage disproportionately on Atlantic herring [1,13] changes in herring dynamics (e.g., growth, distribution, condition) will impact ABFT dynamics in the Gulf of Maine. Our model outcomes relate ABFT distribution to regions of high herring density. Although the model does not account for a larger suite of biophysical variables, it established that ABFT are not randomly distributed on the foraging grounds, and extends findings from a previous study showing that prey (herring) location is the most significant variable predicting weekly ABFT distributions in the Gulf of Maine [23]. Shifts in ABFT distribution coincide with the general rebuilding pattern of Atlantic herring stocks [68,69] in the Gulf of Maine and are currently aligned with regions of historically high herring abundance [68,69]. It is important that future research identifies the mechanisms (bottom-up/top-down) contributing to the regional shifts in prey resources and top predator distribution, especially on the Canadian shelf and offshore regions where ABFT landings ABFT are currently high [64]. Adding biological and ecological insights to the information considered in stock assessments will improve the ability to interpret and predict changes in CPUE or landings for highly migratory species like ABFT, and to reduce uncertainty in stock assessments.
